# Why Is Seed Production So Variable among Individuals? A Ten-Year Study with Oaks Reveals the Importance of Soil Environment

**DOI:** 10.1371/journal.pone.0115371

**Published:** 2014-12-22

**Authors:** Ignacio M. Pérez-Ramos, Cristina Aponte, Luis V. García, Carmen M. Padilla-Díaz, Teodoro Marañón

**Affiliations:** 1 Instituto de Recursos Naturales y Agrobiología de Sevilla (IRNAS), Consejo Superior de Investigaciones Científicas (CSIC), Sevilla, Spain; 2 Department of Forest and Ecosystems Science, University of Melbourne, Melbourne, Australia; INRA - University of Bordeaux, France

## Abstract

Mast-seeding species exhibit not only a large inter-annual variability in seed production but also considerable variability among individuals within the same year. However, very little is known about the causes and consequences for population dynamics of this potentially large between-individual variability. Here, we quantified seed production over ten consecutive years in two Mediterranean oak species – the deciduous *Quercus canariensis* and the evergreen *Q. suber* - that coexist in forests of southern Spain. First, we calibrated likelihood models to identify which abiotic and biotic variables best explain the magnitude (hereafter seed productivity) and temporal variation of seed production at the individual level (hereafter CV_i_), and infer whether reproductive effort results from the available soil resources for the plant or is primarily determined by selectively favoured strategies. Second, we explored the contribution of between-individual variability in seed production as a potential mechanism of satiation for predispersal seed predators. We found that *Q. canariensis* trees inhabiting moister and more fertile soils were more productive than those growing in more resource-limited sites. Regarding temporal variation, individuals of the two studied oak species inhabiting these resource-rich environments also exhibited larger values of CV_i_. Interestingly, we detected a satiating effect on granivorous insects at the tree level in *Q. suber,* which was evident in those years where between-individual variability in acorn production was higher. These findings suggest that individual seed production (both in terms of seed productivity and inter-annual variability) is strongly dependent on soil resource heterogeneity (at least for one of the two studied oak species) with potential repercussions for recruitment and population dynamics. However, other external factors (such as soil heterogeneity in pathogen abundance) or certain inherent characteristics of the tree might be also involved in this process.

## Introduction

There is a growing interest, from an ecological and evolutionary perspective, in understanding the phenomenon of mast-seeding, a very common reproductive strategy in perennial plants that consists of producing occasional large seed crops followed by years of low seed production with a high level of synchronization among neighbouring individuals [1]–[3]. In addition to the high inter-annual variation in seed production, mast-seeding species also exhibit considerable variability among individuals within the same year (e.g. [4]–[8]). However, very little is known about the causes and consequences for population dynamics of this potentially large between-individual variability.

Between-individual variability in seed production could merely respond to variation in available resources for the plant, by analogy with the most parsimonious explanation for masting (resource-tracking hypothesis [2], [9]). Applying this hypothesis to variation among individuals instead of among years (i.e. spatial instead of temporal variation), it would be expected that plants growing in moister and more fertile soils will produce on average larger seed crops than those growing in resource-limited environments. In addition, the propensity for a plant to produce larger or lower seed crops could be also driven by selectively favoured internal plant strategies (e.g. [6], [10]) such as the presence of an endogenous resource-threshold for reproduction with a relative independence of environment influence. However, to what extent reproductive investment tracks resource availability or is primarily determined by selectively favoured strategies is a question that remains largely unexplored, particularly in long-lived species whose reproductive performance varies episodically over time.

Additionally to the potential effect of site quality on the magnitude of reproductive effort (i.e. seed productivity), a high heterogeneity in resource supply could also promote a large variation among individuals in their temporal patterns of reproduction. Kelly and Sork [11] suggested that plants growing in less productive habitats are expected to show higher inter-annual variability in seed production since resource limitation likely increases the time required to accumulate internal resources between subsequent large seed crops. However, very little is known about how different individuals modulate this behaviour as a function of their available resources, and the few studies that have been carried out at the population level have revealed partially inconsistent relationships between mast-seeding and site quality [12]–[14].

A high between-individual variability in seed production could potentially involve important consequences for recruitment and population viability. For instance, large differences among trees in reproductive effort could generate a satiating effect (within a particular year) on those individuals providing the largest seed crops and/or exhibiting the highest inter-annual variability in seed production. This hypothetical satiating effect could be mainly applied to seed predators of low mobility, such as invertebrates (with a limited ability to move between trees), which may be satiated at smaller spatial scale (e.g. at the level of a single tree [11], [15]). Long-term studies on mast-seeding conducted at the individual level are therefore essential to identify the proximate causes of this complex process and infer its potential consequences for the population.

In this paper, we aimed to discern the potential causes and consequences of between-individual variability in reproductive effort (based on a data set of 10 years of seed production) in two oak species co-occurring in Mediterranean forests of southern Spain. First, we calibrated linear and nonlinear likelihood models to explore how different trees invest in seed production (both in magnitude and temporal variability) as a function of their available soil resources (basically soil moisture and fertility). This approach also enabled us to accurately identify which of these soil resources were mainly determining seed productivity and inter-annual variability in seed production at the individual level. Second, we explored the potential contribution of between-individual variability in seed production as a mechanism of satiation (at small spatial scale) on the two main guilds of predispersal seed predators at the study area (vertebrates and granivorous insects [16]).

## Methods

### Ethics Statement

All necessary permits were obtained for the below-described field studies thanks to J. Manuel Fornell Fernández, the Director of “Los Alcornocales” Natural Park.

### Study area and species

The study was conducted in the mixed-oak forests of Aljibe Mountains, near the Strait of Gibraltar, in Southern Spain. Bedrock is predominantly Oligo-Miocene sandstone, which produces acidic, sandy, nutrient-poor soils, although frequently there are interspersed layers of marl sediments, yielding soils richer in clay. Climate is subhumid mediterranean-type, with cool and wet winters, alternating with warm and dry summers. Mean annual temperature ranges from 14.6 to 18.4°C and mean annual rainfall varies from 701 to 1331 mm (mean of 1056 for 15 weather stations over a 20 year period). Vegetation is dominated by evergreen cork oak (*Quercus suber*) forests, mixed with winter-deciduous oaks (*Quercus canariensis*), which are more abundant near streams [17]. Most of the forested area was protected in 1989 as “Los Alcornocales” Natural Park, covering about 1680 km^2^.

The two study *Quercus* species flower in spring (April–May) and acorn development occurs predominantly during summer (from June to September). Seedfall takes place in autumn (from October to February of the following year), *Q. canariensis* acorns being dispersed earlier [16]. Acorns constitute an important part of the diet for many animals. During seed maturation, a variable percentage of acorns are consumed by insects (basically moth or beetle larvae) and some vertebrates such as birds or arboreal rodents [13], [18]–[19]. Once acorns reach the ground, a large part of them are removed and consumed by different predators (e.g. [20]–[21]).

### Sampling design of seed production

In summer 2002, a total of 50 adult, healthy trees of both oak species (20 individuals of *Q. canariensis* and 30 of *Q. suber*) were randomly selected and tagged within the study area. Tree size was characterized by measuring its DBH (diameter at breast height) and the two diameters of the elliptical projection of its crown. All selected trees had a DBH ≥20 cm and presented a dominant or codominant crown position in the forest canopy.

To estimate annual seed production, four circular traps (0.50 m diameter) were randomly placed under the crown of each of the 50 selected trees, trying to avoid overlapping with neighboring plant crowns. Each trap consisted of a plastic mesh attached around an iron ring, which was soldered to an iron rod of 1.5 m in height to avoid acorn removal by rodents (the main acorn consumers at the study area; [22]) after seed drop. Trap content was yearly collected at the end of the seed-drop period (February – March) over ten consecutive years (from 2002 to 2012). Acorns were counted and categorized into four different categories: (1) aborted (not completely or mal-developed seeds, with length <13 mm or diameter <7 mm; *sensu* [23]),(2) insect-infested (having the typical marks caused by oviposition), (3) predispersal vertebrate-predated (identified by signs of rodent gnawing or bird pecking), and (4) mature (sound acorns attaining complete seed size).

In the vicinity of each of the 50 trees (in a 10 m radius plot), we also recorded the number of tree and shrub individuals with DBH>5 cm as a representative measurement of the competition level.

### Characterization of soil environment

Thirteen physicochemical soil properties were assessed beneath the canopy of each of the 50 selected trees. In November 2006, the upper 25 cm of the soil was collected using a soil auger at four different points, that were mixed to produce one composite soil sample beneath each tree (see [24] for further details). Soil samples were dried, crushed and sieved, and the <2-mm fraction was analysed for standard chemical and physical properties [25]. We determined: acidity (with a pH meter), total organic matter (calcination method), total N and C (using an Autoanalyzer LECO), inorganic nitrogen (NH4 and NO3, extracted using 2 M KCl and determined by distillation–titration in a Bran-Luebre Autoanalyzer), extractable P (using the Bray and Kurtz method), available Ca, Mg and K (extracted with ammonium acetate 1 N and determined by atomic absorption spectroscopy), cation exchange capacity (CEC, by the ammonium acetate method), and percentages of sand, silt and clay (using the Bouyoucos hydrometer method). Concentrations of the elements are given on a dry weight basis (105°C). Although these soil properties were quantified only once, they experience temporal changes at a relatively slow rate because they largely depend on a few solid soil phase-related properties (soil particle size, C and N content and CEC), which are strongly stable at the study scale [26].

Soil water content (SWC) was estimated using the gravimetric method. Four soils cores were extracted (using a 3-cm-diameter sampler) and mixed beneath the canopy of each sampling tree at two different depths (0–25 and 25–50 cm). Soil samples were transported to the laboratory in an icebox to determine soil moisture by weighing the fresh and dried (105°C) fractions. These measurements were repeated in spring (May–June), summer (September) and autumn (December) of a whole reproductive cycle (2007). For statistical purposes, the averaged value of the whole soil profile (0–50 cm) was further calculated for each of these seasons.

### Data analyses

#### Components of individual-level variability in seed production

To examine variability patterns of initial (i.e. aborted+mature) and mature seed production for each oak species, we calculated three components related with the process of seed production at the individual level: (i) between-individual variability in seed production (hereafter BIV), calculated as the coefficient of variation (standard deviation/mean) among individuals of the annual (BIV_ann_) or the long-term mean acorn crop (time-averaged values for the 10-year period; BIV_long_); (ii) inter-annual variability in seed production (hereafter CV_i,_ *sensu* [27]), calculated as the coefficient of variation among years of seed production for each of the 50 sampled trees; and (iii) temporal synchrony among trees (rp), calculated as the mean of all pairwise correlations (Pearson's coefficient of correlation) among individual data series [27]. Finally, we also calculated a component of temporal variation at the population level for each of the two studied oak species – CV_p_ - defined as the coefficient of variation of population-averaged values of seed production across years. The number of initial (i.e. aborted and mature) and mature acorns was relativized by m^2^ of basal area, which adjusts for tree size differences.

#### Drivers of seed productivity

To identify which abiotic (particularly soil water content, soil acidity and nutrients) and biotic (density of neighbours) factors could be driving seed productivity at the individual level (number seeds m^-2^ BA), we fitted linear and non-linear models for initial and mature seeds, using maximum likelihood techniques. We tested three alternative functional forms (linear, exponential and Michaelis-Menten), that cover a wide range of possible forms (see equations in S1 Table). We first tested models for each factor and functional form independently, and the best of the three models was compared to a fourth model (the null model) which assumes no effect of any factor. Second, to test for joint limitation (i.e. more than one factor being limiting at once), we fitted bivariate and trivariate models using those factors that had an effect on seed production when evaluated singly. We tried alternative models in which the second factor was added either additively or multiplicatively. This modeling approach is suitable to identify plant responses to abiotic factors at different stages of the regeneration cycle (e.g., [23], [28]–[30]). Models were parameterised with maximum likelihood [31], using a simulating annealing algorithm. Alternative models were compared using the Akaike Information Criterion corrected for small sample sizes (AICc) [32] as a measure of goodness of fit: the lower the AIC value, the better the model. Models with ΔAIC between 0–2 were considered to have equivalent and substantial empirical support [32]. The R^2^ of the regression of observed vs. predicted values was used as an additional measure of the goodness of fit of each alternative model.

#### Drivers of inter-annual variability in seed production

We used the same above-described modelling approach to identify the best predictors of inter-annual variability in seed production at the individual level (CV_i_). All models were implemented using the likelihood package version 1.1 for R and software written specifically for this study in R v 2.5.0 [33]. The modelling approach presented above was also conducted for exploring CV_i_ – environment linkages without considering those trees that exhibited at least one year of null productivity. The comparison between both types of models (i.e. excluding or not trees with zero productivity) enabled us to check whether CV_i_-soil linkages were an artifact of sample size.

#### Potential consequences of between-individual variability in seed production

As a potential consequence of the large between-individual variability in seed production, we finally examined the presence of a ‘satiating’ effect at the tree level by calculating Pearson's coefficients of correlation between the individual seed productivity and the percentages of predated and infested acorns for each of the ten sampling years.

## Results

### Components of individual-level variability in seed production

**Figure 1:**
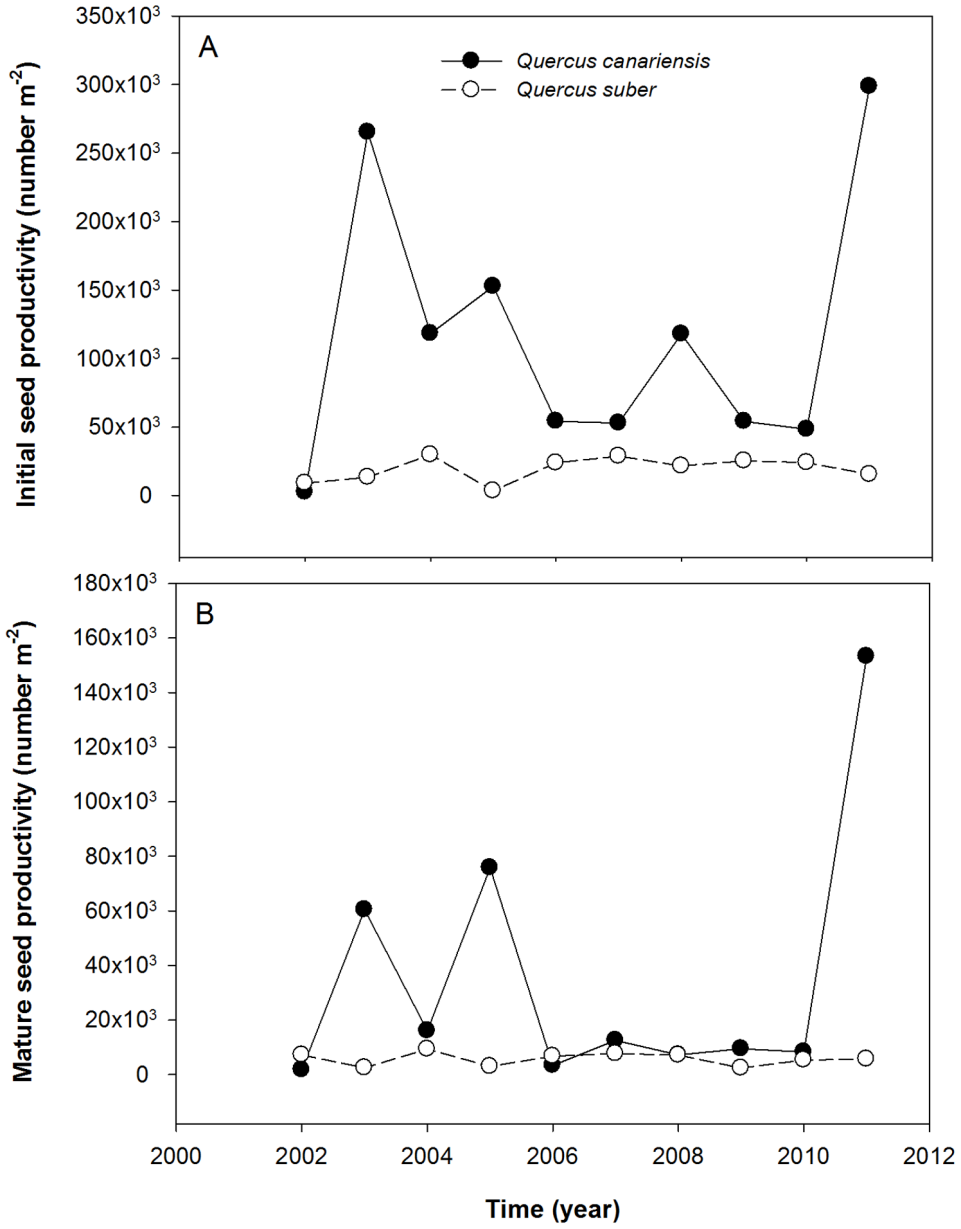
Inter-annual variability in seed production for the two studied oak species (*Quercus canariensis* with solid lines and *Q. suber* with dashed lines) for the entire sampling period (from 2002 to 2012). Two fractions of seed productivity have been represented: initial (including aborted and mature seeds; panel A) and mature acorns (panel B), both of them relativized by m^2^ of tree basal area.

The two studied oak species showed considerable variability among years in seed production, with higher values of CV_p_ and CV_i_ for *Q. canariensis* compared with *Q. suber* (Table 1 and Fig. 1). In addition to this large temporal variability, both species also exhibited a great variability among trees in their time-averaged seed productivity values (BIV_long_) (Table 1 and S1 Fig.). Thus, during the 10-year study period, most of the annual acorn crop was produced by a few trees; for example, about 30% of the sampling trees produced more than 70% of the total number of averaged mature acorns in the two studied species (S1 Fig.). Despite the large between-individual variability in seed production, co-occurring trees exhibited a high level of temporal synchrony (rp), with much higher rp values for mature acorns in the deciduous species (Table 1).

**Table 1:** Components of individual-level variability in seed production (both for initial and mature acorns) for the two studied oak species.

Variability component	BIV_long_	CV_p_	CV_i_	Individual synchrony (Rp)
*Quercus canariensis*				
Initial acorn production	1.0	0.8	1.0	0.54 [18.5%]
Mature acorn production	1.3	1.4	1.4	0.57 [61.1%]
*Quercus suber*				
Initial acorn production	0.9	0.5	0.9	0.50 [17.0%]
Mature acorn production	1.2	0.4	1.2	0.20 [11.9%]

For individual synchrony, the percentage of significant relationships among individuals has been indicated within brackets. BIV_long_ = between-individual variability in seed production using time-averaged values for the 10-year period; CV_p_ = inter-annual variability in seed production at the population level; CV_i_ = inter-annual variability in seed production at the individual level.

In general, both between-individual (BIV_long_) and inter-annual variability (CV_i_ and CV_p_) in seed production increased from initial to mature acorns in the two oak species (Table 1), suggesting that individual mast-seeding could be regulated by processes elapsing from flower fertilization to seed maturation. The only exception was CV_p_ in *Q. suber*, which kept almost constant in both acorn fractions (Table 1). Temporal synchrony (rp) also increased from initial to mature seed production, but only in the case of *Q. canariensis* (Table 1).

### Drivers of seed productivity

**Figure 2:**
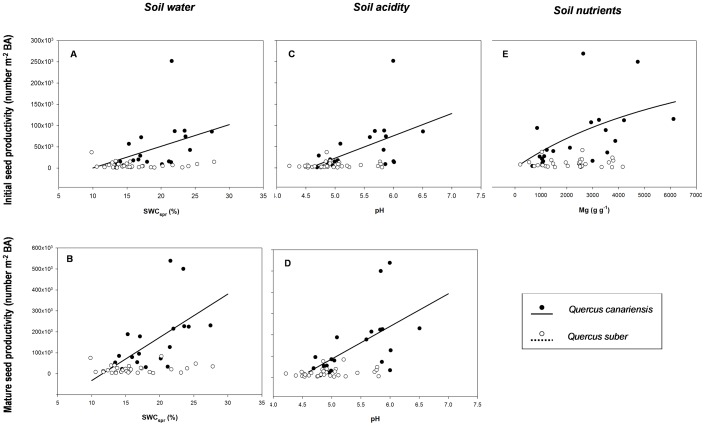
Influence of soil environment (soil water in spring, acidity and nutrients) on initial and mature seed productivity. Values of individual-level seed productivity have been averaged for a time period of 10 years (from 2002 to 2012) and relativized by m^2^ of tree basal area. *Quercus canariensis* has been represented with black symbols and solid lines, whereas *Quercus suber* with white symbols and dashed lines. Lines represent the best-fitted models (details in S1 Table).

In *Q. canariensis*, seed productivity was largely driven by the soil conditions (moisture, acidity and nutrients) present beneath the tree canopy. However this did not occur for the evergreen *Q. suber* (S1 Table). *Q. canariensis* trees inhabiting spring-moister (Fig. 2a and 2b) and less acid soils (Fig. 2c and 2d) produced a higher number of initial and mature acorns. There was also evidence for a positive effect of soil cation concentration on the individual production of initial acorns, which increased linearly with soil K and in saturated way (Michaelis-Menten function) with soil Ca and Mg (Fig. 2e and S1 Table).

### Drivers of inter-annual variability in seed production

**Figure 3:**
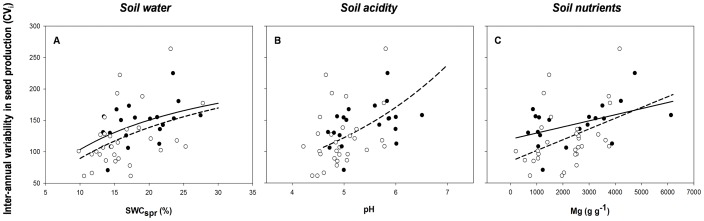
Influence of soil environment (soil water in spring, acidity and nutrients) on inter-annual variability in seed production at the individual level. Values of individual seed production have been averaged for a time period of 10 years (from 2002 to 2012) and relativized by m^2^ of tree basal area. *Quercus canariensis* has been represented with black symbols and solid lines, whereas *Quercus suber* with white symbols and dashed lines. Lines represent the best-fitted models (details in S1 Table).

The temporal component of individual seed production (CV_i_) was also influenced by the same above-mentioned abiotic predictors. On the one hand, trees inhabiting spring-moister (Fig. 3a) and fertile sites (Fig. 3c) showed a higher inter-annual variability in seed production (i.e. larger CV_i_ values, S1 Table). In the specific case of *Q. suber,* CV_i_ also increased exponentially with pH (Fig. 3b). Similar CV_i_ – environment linkages were found after excluding those trees that exhibited at least one year of null productivity (S1 Table), a fact that verifies that these linkages were not an artifact of sample size. The only exception occurred in *Q. suber* for the effect of Mg on CV_i_, which received less empirical support than the null model after excluding the zeros (S1 Table).

### Potential consequences of between-individual variability in seed production

**Figure 4:**
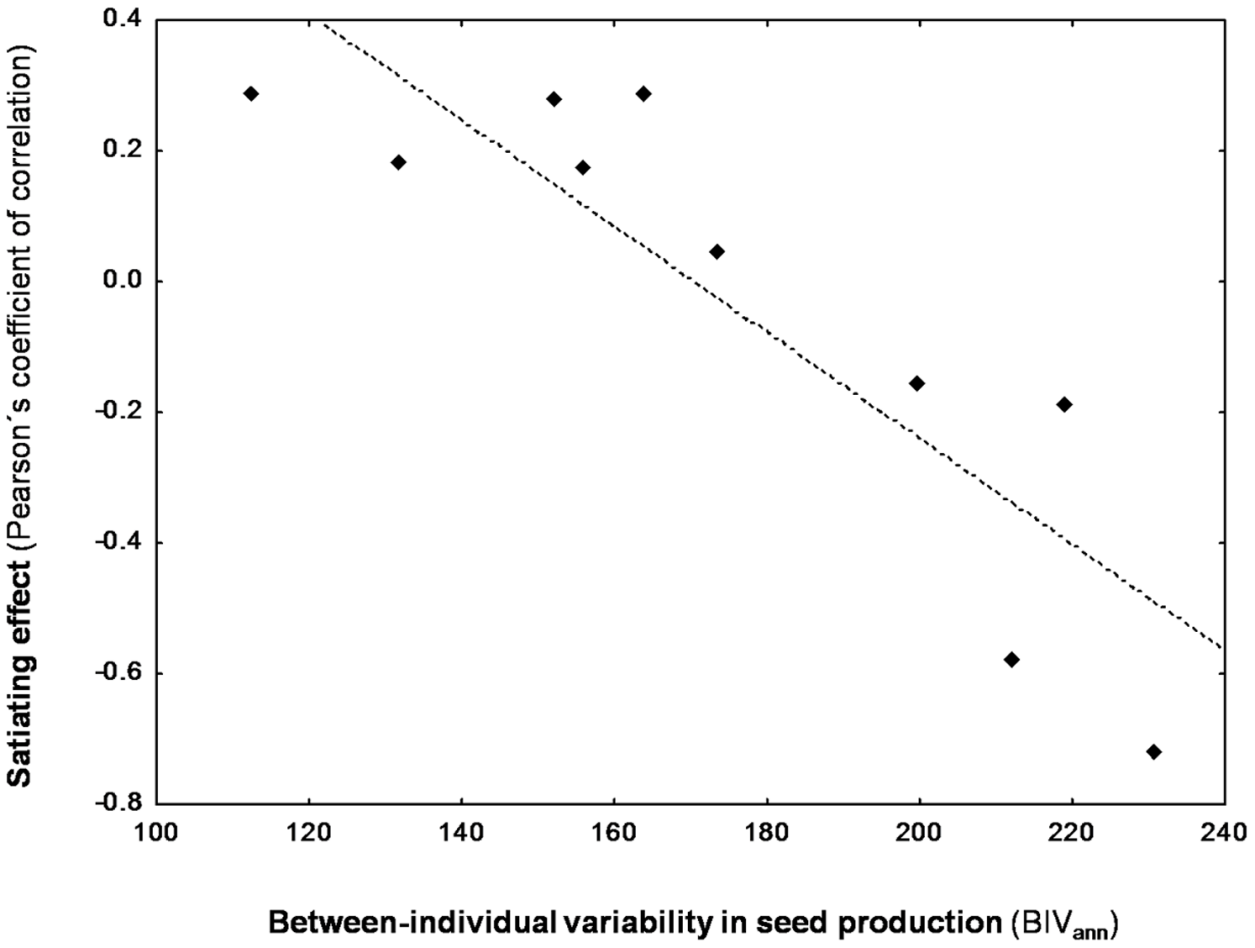
Relationship between inter-individual variability in seed production and the magnitude of a ‘satiating’ effect at the tree level. Between-individual variability in seed production, expressed in terms of coefficient of variation for each of the ten sampling years (BIV_ann_), refers to *Quercus suber* trees. The ‘satiating’ effect has been calculated as the Pearson's coefficient of correlation between the individual productivity of mature acorns and the percentage of them infested by granivorous insects. Black symbols represent each of the ten sampling years.

For the evergreen oak species, we detected a ‘satiating’ effect on the granivorous insects at the tree level, as indicated by the existence of significant negative relationships between the individual production of mature acorns and the percentage of them infested by insects (S2 Table). Interestingly, the magnitude of this ‘satiating’ effect (expressed as the Pearson's coefficient of correlation between the two above-mentioned variables) increased with the between-individual variability in mature seed production (R^2^ = 0.76 and ΔAIC = 9.88; Fig. 4), with significant relationships when BIV_ann_ was over 200. In contrast, a higher individual seed productivity seemed to exercise an opposite (attractive) effect on vertebrate acorn consumers, as indicated by the significant positive relationships detected for some years between the individual production of mature acorns and the percentage of them predated by vertebrates (S2 Table).

The complete data set analysed in this study is available as supporting information in the S1 Data.

## Discussion

### Components of individual-level variability in seed production

Results from our ten-year study showed considerable variability in averaged seed production, not only among years (with CV_p_ values of 1.4 and 0.4 for *Q. canariensis* and *Q. suber*, respectively) but also among individuals within the same population (with seed productivity values varying from 9700 to ≈ 250000 mature acorns by m^2^ of basal area in *Q. canariensis,* and from 630 to ≈ 36700 mature seeds m^-2^ BA in *Q. suber*). These results are in accordance with those reported by previous studies, where most of the annual seed crop was produced by a few specific trees (e.g., [8]–[9]).

Despite the large differences detected in the quantity of acorns produced by different individuals, the temporal pattern of both initial and mature seed production was highly synchronous within populations of *Q. canariensis.* This finding suggests that trees might be responding to some environmental cue that enables them to synchronize their peaks of maximum seed productivity at the same years [34]–[35]. However, temporal synchrony of mature seed production was much lower among individuals of *Q. suber.* The local variability in certain environmental conditions not considered in this study (e.g. radiation, temperature) as well as in some inherent characteristics of the tree (such as those derived of the potential coexistence within populations of individuals with different maturation patterns [36]) might be masking the synchronizing effects of the key environmental drivers that govern seed maturation in the cork oak species.

Our study confirms, therefore, the importance of gathering and analysing data at the individual rather than the population level, as suggested in previous studies (e.g. [34], [37], [38]), and highlights the necessity of considering the between-individual variability in seed production as a quantitatively important component related with the process of mast-seeding.

### Causes of individual-level variability in seed production

We found evidences suggesting that differences among individuals in seed production (both in magnitude and temporal variability) are mainly mediated by soil resource heterogeneity, especially in the deciduous species. These findings support our initial hypothesis that reproductive investment tracks resource availability rather than is mainly governed by selectively favoured internal plant strategies. Such reproductive strategy might provide an alternative way for trees to optimize their performance depending on their available soil resources. Interestingly, the effects of soil resources on seed productivity were only significant for *Q. canariensis,* as explained in detail below.

#### Drivers of seed productivity

Results from our modelling approach showed a strong influence of soil conditions on individual-level seed productivity in the deciduous species. On the one hand, *Q. canariensis* trees inhabiting spring-moister sites produced a higher quantity of seeds than those present in drier areas. Water stress decreases transpiration and photosynthesis rates [39], leading to a significant reduction of growth and storage of energetic reserves, with potentially negative consequences for the internal allocation of resources to reproduction [7]. The influence of water deficit on acorn production has been experimentally demonstrated in recent manipulative studies of rainfall exclusion aimed to evaluate the impact of projected increasing drought on early life-history stages of recruitment [23], [40]. In addition, previous observational studies focused on *Quercus* species have reported than inter-annual fluctuations in seed production are mainly prompted by environmental conditions responsible for soil moisture, such as precipitation levels during specific time periods (e.g. [12]–[14], [36]). However, to our knowledge, there are no previous studies that have identified the main drivers of individual-level seed productivity at a fine spatial scale. The present study is particularly innovative because it describes how different individuals invert more or less in reproduction depending on their local soil conditions (but see [41]). To what extent these between-individual differences in seed productivity resulted from differences in their net productivity (i.e. including both reproductive and vegetative growth) or they were primarily driven by changes in their relative allocation rates to reproduction is a question that merits further research [41].

On the other hand, results from this study demonstrate the relevance of other soil factors different from water, such as soil acidity or nutrients, as key drivers of seed productivity at the individual level. Thus, trees growing in more fertile and less acid soils produced a higher number of initial acorns, presumably mediated by an increase in the number of flowers produced and fertilized. Recent studies have highlighted the crucial role of nutrient availability for the process of mast-seeding, although this time as a mediator of the effect on some synchronizing climatic cues [42]–[43]. The identification of good abiotic predictors for the initial fraction of acorns (including both aborted and mature seeds) in our study suggests a significant role of soil conditions on the processes of flowering, pollination and ovule fertilization. By analogy with the process of mast-seeding, this finding is consistent with previous studies on temporal fluctuations in acorn production (e.g. [14] [44]–[45]) reporting that the strongest correlations with environmental conditions mostly appear for processes occurring before seed maturation. However, the fact that both synchrony and variability among individuals increased from initial to mature seed production indicates that other abiotic factors controlling acorn ripening, such as summer stress, could be also driving these components of individual-level variability in seed production (see similar evidences, but related with temporal fluctuations, in [13] and [23]).

In contrast, soil resource availability did not influence seed productivity in *Q. suber.* For this species, differences among individuals in reproductive investment might be responding to inherent characteristics of the tree or to other factors not considered in this study, such as the differential amount of solar radiation received at the tree crown [44] or the local abundance of soil-borne pathogens responsible for cork oak decline in the study area [46]. Moreover, acorn production in *Q. canariensis* was likely more constrained by resource availability (mainly soil water) than *Q. suber* due to its deciduous leaf habit and its higher sensitivity to water stress (Pérez-Ramos et al. submitted), as indicated by its higher depedence on habitats near streams in the study area [17].

#### Drivers of inter-annual variability in seed production

Regarding the temporal patterns of seed production, results for the two studied oak species were opposite to those expected according to previous hypotheses [11] stating a more pronounced masting in poorer habitats. In our study, trees growing in moister and more fertile soils exhibited the highest inter-annual variability in seed production. According to the ‘resource budget’ model [47]–[48], it would be expected that trees inhabiting these more productive habitats to reach more frequently a high enough level of internal resources exceeding the threshold for reproduction, thus increasing the frequency of mast years and contributing to decrease their individual coefficients of variation among years (CV_i_). However, this common assumption can not be accomplished whether the parameters that define the mast reproductive dynamics of a plant (such as the reproductive threshold or the depletion coefficient) vary with the local availability of soil resources, in agreement with the ‘local adaptation’ hypothesis [49]. This hypothesis states that both the depletion coefficient and the reproductive threshold are genetically determined, appearing a high intraspecific variation for these parameters in response to a high heterogeneity in environmental and ecological conditions. In our case study, we hypothesize that trees growing in resource-rich environments exhibit higher values of reproductive threshold in order to increase their maximum capacity of resource investment for fruiting during the sporadic mast years. According to this hypothesis, trees inhabiting high-productivity habitats invest more energy in reproduction (as indicated by their larger seed crops) but they probably require a higher amount of time to replenish their energy reserves and exceed their enhanced threshold levels. Nevertheless, further studies (at the individual level) considering a broader range of soil conditions than that explored in this study are necessary to determine whether relationships between site quality and mast-seeding fulfil or not the above-mentioned predictions for a wider spatial scale. It would be also interesting to test in the future whether these between-individual differences in CV_i_ are directly affected by soil resource availability or are indirectly mediated by differences in their values of net productivity (which potentially track site quality [41]).

### Potential consequences of inter-individual variability in seed production

Interestingly, we detected a satiating effect on granivorous insects at the level of tree, which was evident in those years where between-individual variability in seed production was higher (with BIV_ann_ values over 200). These results support our initial hypothesis according to which a large variability among individuals in acorn production could generate a satiating effect on low-mobile seed predators at small spatial scale. This satiating effect was only detected for the cork oak species, likely as a consequence of its delayed seed-drop phenology [50], which enabled it to benefit from a reduced seed predation at the expenses of the earlier infestation of its congeneric oak species (see similar results in [51]). Conversely, a higher individual seed productivity seemed to exercise an opposite effect on vertebrate acorn consumers (birds and arboreal rodents), trees bearing a higher quantity of acorns attracting a larger number of these generalist, predispersal acorn predators. The opposite effects of big seed crops on invertebrate (less mobile) and vertebrate (more mobile) seed predators has been discussed in previous studies, but referred to inter-annual fluctuations [38], [52].

These complex interactions between insects, vertebrates and *Quercus* species could have important repercussions for oak recruitment. Thus, in plant populations where the overabundance of vertebrate seed predators seriously constrain natural regeneration, such as the studied oak forest sites [22], the attractive effect provided by the most productive trees could counteract the satiating effect exercised on the process of seed infestation. However, in forest sites where the pressure of these two guilds of seed predators is more balanced, the risk of seed predation could be reduced as a consequence of these two sources of variability. Thus, between-individual variability in seed production might locally satiate invertebrates, whereas inter-annual variability in seed crop sizes might reduce pre- and post-dispersal acorn consumption by vertebrates in masting years.

## Conclusions

In summary, evidence presented here indicate that between-individual variability in seed production is mainly a direct consequence of the heterogeneous soil environment, at least for one of the two studied oak species (*Q. canariensis*). Although our findings agree most closely with our initial hypothesis, we can not conclude that differences among individuals in seed production are only prompted by soil resources available for the plant. Thus, the apparent independence of soil conditions for reproduction in *Q. suber* suggests that other external factors (such as soil heterogeneity in pathogen abundance) or certain inherent characteristics of the tree might be also driving the propensity for a tree to produce larger or lower seed crops at regular periods of time. Further studies on individual plants in other tree species and systems are thereby essential to discern the causes and consequences of this often ignored component of variability in seed production, with potentially important repercussions for recruitment and dynamics of tree populations.

## Supporting Information

S1 Figure **Averaged seed productivity of the 50 sampling trees (20 of** ***Quercus canariensis*** **- panel A - and 30 of** ***Q. suber*** **- panel B -).** Two fractions of seed productivity have been represented: initial (including aborted and mature seeds) and mature acorns, both of them relativized by m^2^ of tree basal area. Values of seed productivity have been averaged for a time period of 10 years (from 2002 to 2012). Trees have been re-ordered (from C-1 to C-20 for *Q.canariensis*, and from S-1 to S-30 for *Q. suber*) based on their ten-year averaged values of initial seed productivity. Vertical bars denote standard-error values.
(TIF)

S1 Table **Summary of the best-fitted models analyzing how the components of individual-level variability in seed production (productivity of initial and mature seeds, and inter-annual variability in seed production) respond to soil factors, for the two studied oak species.** CV_i_ – environment linkages were also conducted without considering those trees that exhibited at least one year of null productivity in order to verify that these linkages were not an artifact of sample size. Only the models with better empirical support than null are shown, ranked from best to poorest fits. The best-supported model and their equivalents (ΔAIC<2) have been highlighted with bold letters for each component. The signs of the relationships (positive or negative) between each dependent variable and the selected soil predictors are also indicated. Model Forms: LIN, linear model; EXP, exponential model; MM, Michaelis-Menten model; null, null model.
(DOC)

S2 Table **Pearson's coefficients of correlation between individual productivity of mature acorns and percentages of seed infestation (by granivorous insects) and predation (by vertebrates) for each of the ten sampling years (from 2002 to 2012).** The significance level is indicated as follows: ***P<0.001; **P<0.01; *P<0.05. Significant values of “r” have been highlighted with bold letters.
(DOC)

S1 Data **Data set of seed productivity, interannual variability in seed production (CV_i_), percentages of the different seed categories (i.e. abortions, vertebrate-predated and insect-infested seeds) and soil environment beneath the canopy (moisture, acidity, texture and main nutrients) of the 50 trees considered in the present study.** Values of individual-level seed productivity have been averaged for a time period of 10 years (from 2002 to 2012) and relativized by m^2^ of tree basal area.
(DOC)
