# Supplementary figures and images for: Why Is Seed Production So Variable among Individuals? A Ten-Year Study with Oaks Reveals the Importance of Soil Environment
Source: PLoS One. 2014 Dec 22;9(12):e115371. doi: 10.1371/journal.pone.0115371 (PMC4274023; doi:10.1371/journal.pone.0115371)

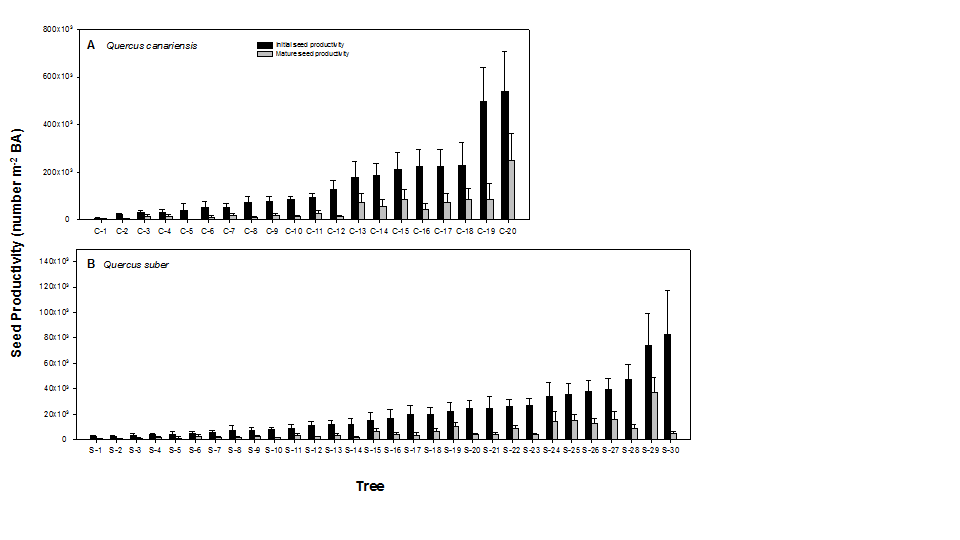

Supplement: S1 Figure — Averaged seed productivity of the 50 sampling trees (20 of Quercus canariensis - panel A - and 30 of Q. suber - panel B -). Two fractions of seed productivity have been represented: initial (including aborted and mature seeds) and mature acorns, both of them relativized by m2 of tree basal area. Values of seed productivity have been averaged for a time period of 10 years (from 2002 to 2012). Trees have been re-ordered (from C-1 to C-20 for Q.canariensis, and from S-1 to S-30 for Q. suber) based on their ten-year averaged values of initial seed productivity. Vertical bars denote standard-error values. (TIF) [file pone.0115371.s001.tif]
