# Supplementary material for: Why Is Seed Production So Variable among Individuals? A Ten-Year Study with Oaks Reveals the Importance of Soil Environment
Source: PLoS One. 2014 Dec 22;9(12):e115371. doi: 10.1371/journal.pone.0115371 (PMC4274023; doi:10.1371/journal.pone.0115371)
Supplement: S1 Table — Summary of the best-fitted models analyzing how the components of individual-level variability in seed production (productivity of initial and mature seeds, and inter-annual variability in seed production) respond to soil factors, for the two studied oak species. CVi – environment linkages were also conducted without considering those trees that exhibited at least one year of null productivity in order to verify that these linkages were not an artifact of sample size. Only the models with better empirical support than null are shown, ranked from best to poorest fits. The best-supported model and their equivalents (ΔAIC<2) have been highlighted with bold letters for each component. The signs of the relationships (positive or negative) between each dependent variable and the selected soil predictors are also indicated. Model Forms: LIN, linear model; EXP, exponential model; MM, Michaelis-Menten model; null, null model. (DOC) [file pone.0115371.s002.doc]

**Table S1.**

|  |  |  |  |  |  |  |  |
| --- | --- | --- | --- | --- | --- | --- | --- |
|  | **Dependent variable** | **Abiotic factors** | **Model** | **Relation** | **R^2^** | **AIC** | **∆AIC** |
| *QUERCUS CANARIENSIS* | *Initial seed productivity (nb m^-2^ BA)* | **SWC_spr_** | **LIN** | **+** | **0.35** | **162.00** | **0.00** |
|  |  | **pH** | **LIN** | **+** | **0.33** | **162.77** | **0.77** |
|  |  | **Mg** | **MM** | **+** | **0.31** | **163.38** | **1.38** |
|  |  | Ca | MM | + | 0.28 | 164.13 | 2.13 |
|  |  | K | LIN | + | 0.25 | 164.98 | 2.98 |
|  |  |  | null |  |  | 167.93 | 5.93 |
|  |  |  |  |  |  |  |  |
|  | *Mature seed productivity (nb m^-2^ BA)* | **pH** | **LIN** | **+** | **0.26** | **127.13** | **0.00** |
|  |  | **SWC_spr_** | **LIN** | **+** | **0.23** | **127.89** | **0.76** |
|  |  |  | null |  |  | 130.32 | 3.19 |
|  |  |  |  |  |  |  |  |
|  | *Inter-annual variability in seed production (CV_i_)* | **Ca** | **LIN** | **+** | **0.30** | **195.23** | **0.00** |
|  |  | **Mg** | **LIN** | **+** | **0.23** | **197.20** | **1.98** |
|  |  | SWC_spr_ | MM | + | 0.21 | 197.48 | 2.25 |
|  |  |  | null |  |  | 199.51 | 4.28 |
|  |  |  |  |  |  |  |  |
|  | *Inter-annual variability in seed production (CV_i_) - excluding zeros -* | **Ca** | **LIN** | **+** | **0.44** | **93.21** | **0.00** |
|  |  | **Mg** | **LIN** | **+** | **0.41** | **93.75** | **0.54** |
|  |  | SWCspr | LIN | + | 0.29 | 95.39 | 2.18 |
|  |  |  | null |  |  | 97.48 | 4.27 |
|  |  |  |  |  |  |  |  |
| *QUERCUS SUBER* | *Initial seed productivity (nb m^-2^ BA)* |  | null |  |  | 76.50 |  |
|  |  |  |  |  |  |  |  |
|  | *Mature seed productivity (nb m^-2^ BA)* |  | null |  |  | 76.50 |  |
|  |  |  |  |  |  |  |  |
|  | *Inter-annual variability in seed production (CV_i_)* | **SWC_spr_** | **MM** | **+** | **0.18** | **316.33** | **0.00** |
|  |  | **Mg** | **LIN** | **+** | **0.16** | **317.00** | **0.67** |
|  |  | **pH** | **EXP** | **+** | **0.14** | **317.71** | **1.38** |
|  |  |  | null |  |  | 319.71 | 3.38 |
|  |  |  |  |  |  |  |  |
|  | *Inter-annual variability in seed production (CV_i_) - excluding zeros -* | **SWC_spr_** | **MM** | **+** | **0.19** | **139.31** | **0.00** |
|  |  | **pH** | **EXP** | **+** | **0.17** | **139.63** | **0.32** |
|  |  |  | null |  |  | 142.03 | 2.72 |
|  |  |  |  |  |  |  |  |

Note: The equations of the different functions fitted in the models calibrated for this study are:

1. Linear aditive: *a + bFactorAi + cFactorBi*
2. Linear multiplicative: *a + bFactorAi * cFactorBi*
3. Exponential aditive: *ae ^(bFactorAi+ cFactorBi)^*
4. Exponential multiplicative: *ae ^(bFactorAi *cFactorBi)^*
5. Michaelis- Menten multiplicative:

*aFactorAi * FactorBi*

*─────────────────────────*─────────

*(a/b + FactorAi) * (a/c + FactorBi)*

where a, b, and c are parameter estimates that maximized the likelihood function, and *Factors Ai* and *Bi* are the selected predictor abiotic variables for each individual “*i*”.
