# Supplementary material for: Why Is Seed Production So Variable among Individuals? A Ten-Year Study with Oaks Reveals the Importance of Soil Environment
Source: PLoS One. 2014 Dec 22;9(12):e115371. doi: 10.1371/journal.pone.0115371 (PMC4274023; doi:10.1371/journal.pone.0115371)
Supplement: S2 Table — Pearson's coefficients of correlation between individual productivity of mature acorns and percentages of seed infestation (by granivorous insects) and predation (by vertebrates) for each of the ten sampling years (from 2002 to 2012). The significance level is indicated as follows: ***P<0.001; **P<0.01; *P<0.05. Significant values of “r” have been highlighted with bold letters. (DOC) [file pone.0115371.s003.doc]

**Table S2.**

|  |  |  |  |  |  |  |  |  |  |  |
| --- | --- | --- | --- | --- | --- | --- | --- | --- | --- | --- |
|  | Mature seed productivity (number m^-2^) | | | | | | | | | |
|  | 2002/2003 | 2003/2004 | 2004/2005 | 2005/2006 | 2006/2007 | 2007/2008 | 2008/2009 | 2009/2010 | 2010/2011 | 2011/2012 |
| *Quercus canariensis* |  |  |  |  |  |  |  |  |  |  |
| Acorn predation (%) | - | -0.13 | -0.08 | 0.02 | -0.08 | **0.93***** | -0.11 | 0.47 | -0.28 | **0.46*** |
| Acorn infestation (%) | -0.45 | -0.13 | 0.28 | 0.23 | 0.34 | 0.49 | -0.36 | -0.27 | 0.07 | -0.41 |
|  |  |  |  |  |  |  |  |  |  |  |
| *Quercus suber* |  |  |  |  |  |  |  |  |  |  |
| Acorn predation (%) | 0.18 | -0.30 | -0.09 | **0.76***** | -0.14 | -0.31 | -0.47 | -0.10 | -0.29 | -0.33 |
| Acorn infestation (%) | -0.19 | 0.18 | -0.15 | **-0.58*** | 0.28 | 0.05 | **-0.72*** | 0.29 | 0.17 | 0.29 |
|  |  |  |  |  |  |  |  |  |  |  |
|  |  |  |  |  |  |  |  |  |  |  |
