# Supplementary material for: Why Is Seed Production So Variable among Individuals? A Ten-Year Study with Oaks Reveals the Importance of Soil Environment
Source: PLoS One. 2014 Dec 22;9(12):e115371. doi: 10.1371/journal.pone.0115371 (PMC4274023; doi:10.1371/journal.pone.0115371)
Supplement: S1 Data — Data set of seed productivity, interannual variability in seed production (CVi), percentages of the different seed categories (i.e. abortions, vertebrate-predated and insect-infested seeds) and soil environment beneath the canopy (moisture, acidity, texture and main nutrients) of the 50 trees considered in the present study. Values of individual-level seed productivity have been averaged for a time period of 10 years (from 2002 to 2012) and relativized by m2 of tree basal area. (DOC) [file pone.0115371.s004.doc]

**Data S1.**

**Appendix 4.** (Continued)
